# Association of left atrial volume index and all-cause mortality in patients referred for routine cardiovascular magnetic resonance: a multicenter study

**DOI:** 10.1186/s12968-018-0517-0

**Published:** 2019-01-07

**Authors:** Mohammad A. Khan, Eric Y. Yang, Yang Zhan, Robert M. Judd, Wenyaw Chan, Faisal Nabi, John F. Heitner, Raymond J. Kim, Igor Klem, Sherif F. Nagueh, Dipan J. Shah

**Affiliations:** 1 Department of Cardiology, Houston Methodist Hospital, 6550 Fannin St., Suite 677, Smith Tower, Houston, TX 77030 USA; 2 Department of Cardiology, New York Methodist Hospital, New York, USA; 3 Department of Cardiology, Duke University, Durham, North Carolina USA; 4 Department of Biostatistics, University of Texas Health Science Center, Houston, TX USA; 5 Department of Medicine, Flushing Hospital Medical Center, Flushing, New York, USA

**Keywords:** Left atrial volume, Mortality, Cardiac magnetic resonance, Biplane area-length method

## Abstract

**Background:**

Routine cine cardiovascular magnetic resonance (CMR) allows for the measurement of left atrial (LA) volumes. Normal reference values for LA volumes have been published based on a group of European individuals without known cardiovascular disease (CVD) but not on one of similar United States (US) based volunteers. Furthermore, the association between grades of LA dilatation by CMR and outcomes has not been established. We aimed to assess the relationship between grades of LA dilatation measured on CMR based on US volunteers without known CVD and all-cause mortality in a large, multicenter cohort of patients referred for a clinically indicated CMR scan.

**Method:**

We identified 85 healthy US subjects to determine normal reference LA volumes using the biplane area-length method and indexed for body surface area (LAVi). Clinical CMR reports of patients with LA volume measures (*n* = 11,613) were obtained. Data analysis was performed on a cloud-based system for consecutive CMR exams performed at three geographically distinct US medical centers from August 2008 through August 2017. We identified 10,890 eligible cases. We categorized patients into 4 groups based on LAVi partitions derived from US normal reference values: Normal (21–52 ml/m^2^), Mild (52–62 ml/m^2^), Moderate (63–73 ml/m^2^) and Severe (> 73 ml/m^2^). Mortality data were ascertained for the patient group using electronic health records and social security death index. Cox proportional hazard risk models were used to derive hazard ratios for measuring association of LA enlargement and all-cause mortality.

**Results:**

The distribution of LAVi from healthy subjects without known CVD was 36.3 ± 7.8 mL/m^2^. In clinical patients, enlarged LA was associated with older age, atrial fibrillation, hypertension, heart failure, inpatient status and biventricular dilatation. The median follow-up duration was 48.9 (IQR 32.1–71.2) months. On univariate analyses, mild [Hazard Ratio (HR) 1.35 (95% Confidence Interval [CI] 1.11 to 1.65], moderate [HR 1.51 (95% CI 1.22 to 1.88)] and severe LA enlargement [HR 2.14 (95% CI 1.81 to 2.53)] were significant predictors of death. After adjustment for significant covariates, moderate [HR 1.45 (95% CI 1.1 to 1.89)] and severe LA enlargement [HR 1.64 (95% CI 1.29 to 2.08)] remained independent predictors of death.

**Conclusion:**

LAVi determined on routine cine-CMR is independently associated with all-cause mortality in patients undergoing a clinically indicated CMR.

## Background

Left atrial (LA) dilation is associated with various cardiac disorders, such as valvular heart diseases [1, 2], left ventricular (LV) systolic [3] or diastolic dysfunction [4], obstructive sleep apnea [5, 6], and atrial fibrillation [7–9]. LA enlargement is a risk marker for the future development of atrial fibrillation [8, 10], and is associated with heart failure hospitalizations, stroke [11, 12], and death [13–15].

Cardiovascular magnetic resonance imaging (CMR) is the gold standard for measuring cardiac chamber volumes due to its superior accuracy and precision compared to other imaging modalities [16–18]. Although reference LA chamber size values have been defined from multiple studies, the process requires tomographic slices through the atrial chambers that is not routinely used in clinical practice, as it can be time-consuming and challenging for clinical patients with dyspnea. Additionally, these reference values are based off of studies using European subjects and there have been no known studies comprising healthy subjects without known cardiovascular diseases (CVD) from the United States that may vary in body habitus and ethnicity. While there are limited data exploring the association of CMR-derived LA volume with patient outcomes in selected cohorts [19–22], there is a lack of data on the association of LA volumes by CMR, categorized by worsening grades of LA enlargement, with all-cause mortality in a large general patient cohort. We aimed to measure the association of all-cause mortality with different severity grades LA enlargement, derived from using routine clinically available CMR images using the area-length method in a large patient population referred for CMR.

Our first objective was to define normal LA volumes for healthy US subjects by CMR based upon routinely acquired cardiac imaging planes using the biplane area-length method. In our second objective, we explored the association of LA size with all-cause mortality in a large cohort of patients referred for clinical CMR. We further assessed for the persistence of such an association after adjusting for clinically relevant variables, bi-ventricular ejection fraction (EF), and LV myocardial scar.

## Methods

### Part A: Healthy subjects

Healthy volunteer subjects (*n* = 85) without any known CVD were recruited from Houston Methodist between October 2008–July 2017 to undergo CMR for assessment of LA volumes. LA volumes were calculated using the biplane area-length method. Height, weight, blood pressure and heart rate were obtained from each subject at the time of the scan. Body surface area (BSA) was calculated using the Mosteller formula [23] for indexing of CMR parameters to body size. The mean LA volume indexed to BSA (LAVi) with the standard deviation was calculated for healthy volunteer subjects. Normal reference range was defined as 2 standard deviations above and below the mean LAVi.

### Part B: Clinical patient cohort

We acquired patient data from our data coordinating center, which uses a cloud-based database (CloudCMR, www.cloudCMR.com) containing de-identified searchable data from consecutive patients with full DICOM datasets from three geographically distinct medical centers in the United States. All data fields were derived from CMR reports that had been analyzed and electronically signed by board-certified physicians with Level III CMR training. LA volumes in all patients were also measured using the biplane area-length method in the same fashion used in the healthy volunteer subject group. We acquired 11,613 unique patient cases from August 2008 through August 2017 for review. We excluded patients with age less than 18 years (*n* = 171) and missing BSA (*n* = 177).

Patients were classified into “Normal”, “Mild”, “Moderate” or “Severe” LAVi groups based upon the severity of LA enlargement. Patients in the “Normal” group had LAVi that fell within the normal reference range which was derived using data from the healthy subject cohort. Using the receiver operating characteristics (ROC) analysis for risk of death in patients with LAVi greater than the upper limit of “Normal”, an optimal cutoff value for LAVi was generated. Patients with LAVi greater than this cutoff value were categorized in the “Severe” group. A midpoint was then identified between the upper limit of “Normal” and the lower limit of “Severe” (ROC generated cutoff value). Patients with LAVi between the upper limit of “Normal” and the midpoint were categorized in the “Mild” group, while patients with LAVi between the midpoint and the lower limit of “Severe” were categorized in the “Moderate” group. The method we used to categorize severity of LA enlargement has previously been published [24].

Due to concerns of foreshortening, instances with calculated LAVi more than 2 standard deviations below the normal reference mean were excluded (*n* = 375). Our final study population consisted of 10,890 patients.

### Clinical data

We acquired demographic, basic anthropometric, clinical and CMR measured parameters through CloudCMR. Demographic information and relevant medical history were collected from the patients prior to the scan. A registered nurse assigned to the CMR lab measured height, weight, heart rate, and blood pressure of each patient. History of medication use and CVD risk factors such as diabetes mellitus, hypertension, dyslipidemia, family history of coronary artery disease, and history of smoking were self-reported by patients. A plasma creatinine level was measured using the i-STAT® analyzer or through the respective institution’s laboratory for patients scheduled to receive gadolinium. Estimated glomerular filtration rate (eGFR) was calculated through “The Modification of Diet in Renal Disease” study equation [25]. The cardiac rhythm for each patient was noted during the scan.

### CMR parameters

Participants were scanned on either 1.5 Tesla or 3.0 Tesla magnetic resonance CMR scanners (Avanto and Verio scanners respectively, Siemens Healthineers, Erlangen, Germany). We used balanced steady-state free precession (bSSFP) cine images to acquire standard 4-chamber and 2-chamber views of the left heart. Ventricular volumes were determined by manually tracing endocardial borders in serial short-axis images from the base of the heart to the apex in end-systole and end-diastole. Image acquisition parameters using bSSFP were: slice thickness of 6.0 mm with a 4-mm gap; in plane resolution of ~ 1.5 × 1.5 × 2.1 mm. Repetition time and echo time was tailored for each patient to achieve 25 to 30 cardiac phases per cardiac cycle. In patients undergoing contrast CMR, late gadolinium enhancement (LGE) images were obtained 5–15 min after the administration of an intravenous contrast agent at a dose of 0.15 to 0.20 mmol/kg.

#### Post processing

**Fig. 1:**
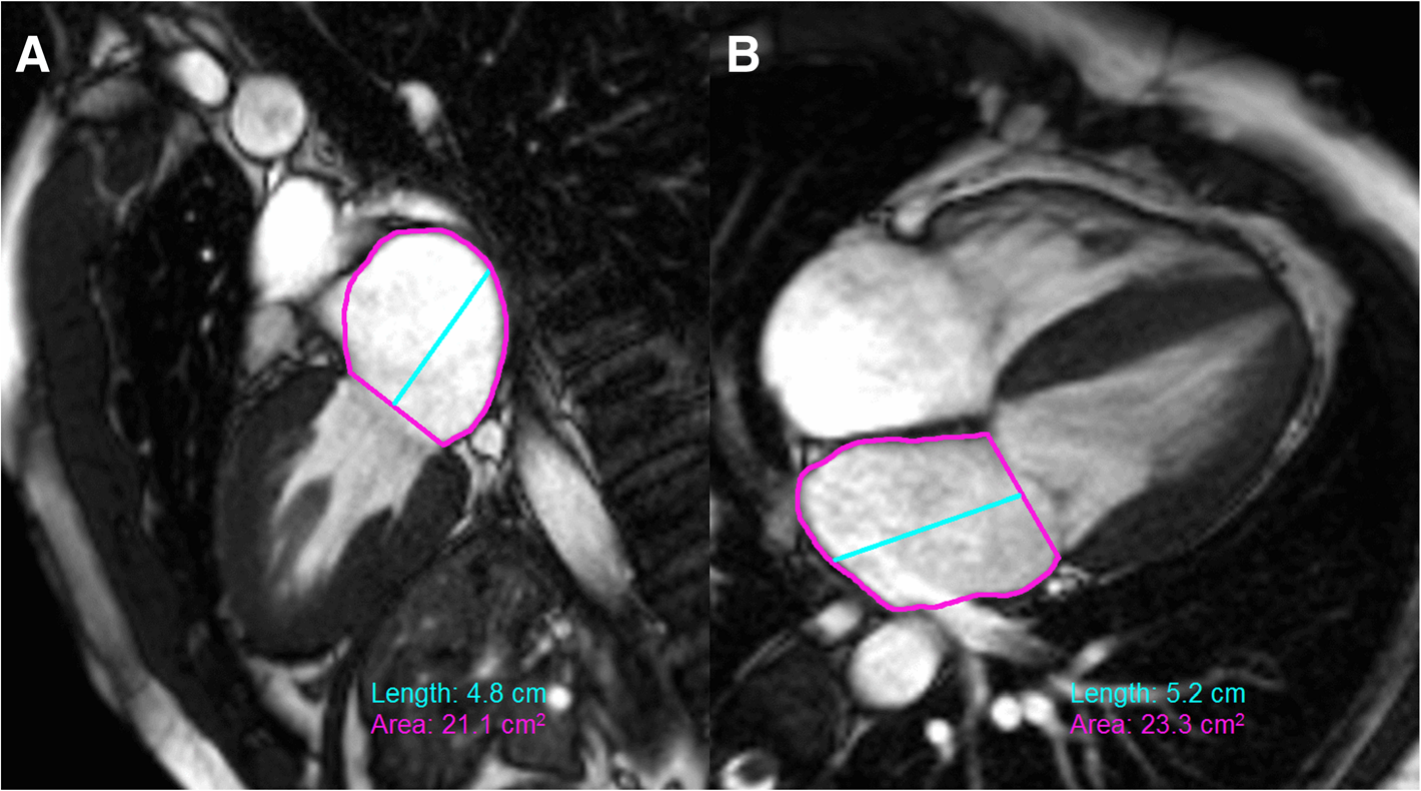
Two (**a**) and four (**b**) chamber CMR left ventricular view tracing of left atrium (LA). The LA appendage and pulmonary veins are excluded from area planimetry. Length is drawn as a perpendicular line from mid-point of the straight line connecting the mitral annulus

Determination of end-systolic and end-diastolic phases was assessed visually with the frames having maximum and minimum LV cavity area as end-diastole and end-systole, respectively. After tracing the epicardial LV borders, LV myocardial mass was assessed by measuring the area in each of the short-axis slices between the endocardial and epicardial tracing, multiplied by 1.04 ml/g. LV papillary muscles were traced and therefore counted towards the LV mass and not LV volume. Maximal LA volume was determined using the biplane area-length method in 4- and 2-chamber LV long axis views at LV end-systole (referenced by the time frame prior to opening of the mitral valve). We excluded the LA appendage and pulmonary veins from the LA tracing in 4- and 2- chamber LV views due to anatomic variability between patients and to preserve reproducibility as it is not always captured in a standard 2- and 4- chamber LV views (Fig. 1).

The formula for calculating the LA volume using Biplane area-length method is given as follows:

$$ \frac{8}{3\uppi}\times \left(\frac{\mathrm{A}4\mathrm{c}\times \mathrm{A}2\mathrm{c}}{\mathrm{L}}\right) $$

where *A4c* and *A2c* corresponds to LA areas in 4- and 2- chamber views respectively, and *L* corresponds to the shortest long-axis length measured in either views [26].

The LA volume was then divided by BSA to index for body size; LAVi.

### Intra-observer and inter-observer reproducibility

For intra-observer reproducibility, observer A (MAK) measured LA volumes of all the healthy subjects. Observer A then re-measured the LA volumes 3 months later blinded to previous measurements. For inter-observer reproducibility, observer B (YZ) measured the LA volumes in all CMR studies of healthy subjects independently. For inter-site reproducibility, observer A re-measured LA volumes of 30 randomly selected cases from the patient cohort, from each site (*n* = 90). To demonstrate reproducibility of measuring LAVi, all analyses were conducted using volumes indexed to BSA.

### Outcome measures

The outcome was defined as all-cause mortality for our cohort. Mortality was ascertained in the patient cohort on September 2017, by accessing electronic health records of patients and by matching patients to the social security death index (SSDI) database prior to anonymization and upload to CloudCMR. The median duration for the patient cohort from date of scan to ascertainment for an event was 48.9 (interquartile range 32.1–71.2) months.

### Statistical analysis

Statistical analyses were performed using Stata 14.2 (StataCorp LP, College Station, Texas, USA). A *P*-value of < 0.05 was considered significant. We compared baseline characteristics, clinical data, and CMR data between groups with increasing severity of LA volume enlargement. Continuous variables were described as medians (interquartile ranges). All relevant continuous variables were found not to be normally distributed by a statistically significant Shapiro-Wilk test result; hence the Kruskal-Wallis test was used for comparison testing among multiple groups. The categorical variables were reported as proportions, which were compared among groups using the Chi squared (χ^2^) test. The sensitivity and specificity of LAVi for determining the risk of death in patients with LAVi greater than the upper limit of “Normal” was confirmed by ROC analysis. The optimal cutoff value for LAVi was generated using the Youden’s J statistic.

To assess the association of mortality with categorical grade of LA enlargement severity and using LAVi as a continuous variable, univariate and multivariate Cox proportional hazard risk models were used to derive hazard risk ratios. The multivariate models included the categorical grade of LA enlargement severity or continuous LAVi as an independent predictor variable, along with other predictors of mortality that showed statistical significance on univariate analyses. Separately, continuous LAVi was examined as a predictor of mortality using restricted cubic spline regression model to understand the hazard risk ratio at any given LAVi value. The benefit of using restricted cubic spline allows us to demonstrate a potentially non-linear relationship between LAVi and all-cause mortality. The restricted cubic spline curve was made with 4 knots based on LAVi quantiles.

Intraclass correlation coefficients (ICC) were calculated to assess the intra-observer and inter-observer reproducibility for LA volume measurements. ICC values from 0.75 to 1.0 were considered excellent.

## Results

**Fig. 2:**
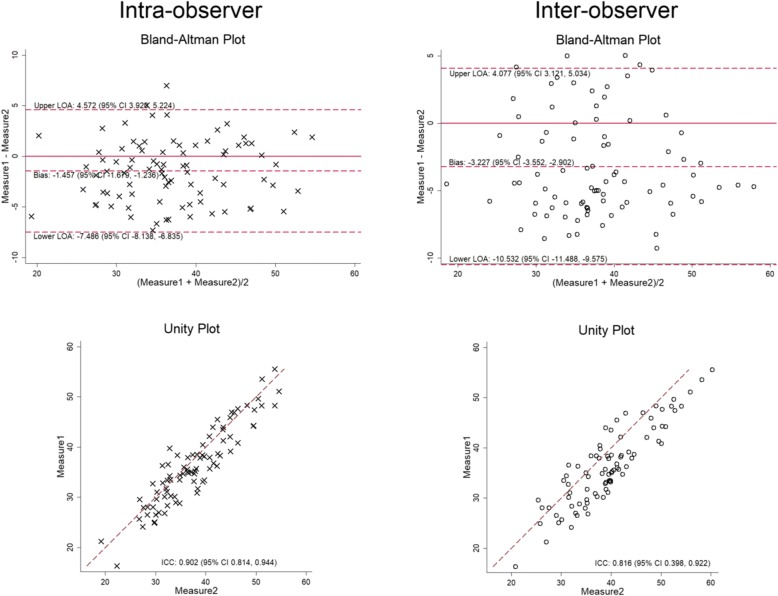
Bland-Altman and unity plots for intra-observer and inter-observer reproducibility analysis of LA volume index (LAVi) measurement

In our healthy cohort we derived a mean LAVi of 36.3 (standard deviation [SD] 7.8) mL/m^2^ which was similar between men (36.5 (SD 7.8) mL/m^2^) and women (36.1 (SD 7.7) mL/m^2^). Median age was 38 years (30, 46 Interquartile range [IQR]) with 41% of the participants being females. Males tended to have larger body surface area and absolute LA volumes than females. The baseline characteristics of our healthy subjects are described (Table 1). Based on the ICC value of 0.90 for intra-observer, and 0.82 for inter-observer; LAVi measurement reproducibility was excellent (Fig. 2). Inter-site reproducibility was also excellent between the central reader (observer A) and the three different sites (Overall [*n* = 90] ICC: 0.94 [95% confidence interval (CI) 0.91, 0.96] and Bias: 1.84 [95% limits of agreement (LOA) 16.45, − 12.8], Site 1 [*n* = 30] ICC: 0.95 [95% CI 0.9, 0.98] and Bias: 2.6 [95% LOA 16.8, − 11.6], Site 2 [*n* = 30] ICC: 0.94 [95% CI 0.78, 0.98] and Bias: 5.2 [95% LOA 19.2, − 8.8], and Site 3 [*n* = 30] ICC: 0.92 [95% CI 0.83, 0.96] and Bias: -2.3 [95% LOA 9.6, − 14.1]).

**Table 1:** Baseline characteristics of healthy subject cohort

Variable ^a^	Total (*n* = 85)	Females (*n* = 35)	Males (*n* = 50)
Age (years)	39 ± 12	39 ± 14	39 ± 10
Race			
White (%)	51%	56%	47%
Black (%)	9%	17%	4%
Asian (%)	26%	23%	29%
Other (%)	14%	5%	20%
Anthropometric Indices			
Height (cm)	171 ± 10.2	162 ± 6.4	177 ± 8.5
Weight (kg)	75.8 ± 21	63.8 ± 16.2	84.7 ± 19.4
Body Surface Area (m^2^)	1.87 ± 0.28	1.67 ± 0.18	2.01 ± 0.25
Heart Rate (bpm)	74 ± 11	75 ± 13	74 ± 11
Systolic Blood Pressure (mmHg)	123 ± 13	123 ± 14	123 ± 13
Diastolic Blood Pressure (mmHg)	78 ± 12	75 ± 14	79 ± 12
Left Atrial Indices			
Diameter (cm)	3.2 ± 0.5	2.9 ± 0.5	3.4 ± 0.5
Area 4 Chamber (cm^2^)	19.7 ± 4.0	18.1 ± 2.7	20.8 ± 4.4
Area 4 Chamber Indexed (cm^2^/m^2^)	10.6 ± 1.7	10.9 ± 1.6	10.4 ± 1.7
Length 4 Chamber (cm)	4.9 ± 0.7	4.6 ± 0.6	5 ± 0.7
Area 2 Chamber (cm^2^)	18.1 ± 4.2	16.6 ± 3.8	19.1 ± 4.3
Area 2 Chamber Indexed (cm^2^/m^2^)	9.7 ± 1.9	10 ± 2.0	9.5 ± 1.8
Length 2 Chamber (cm)	4.5 ± 0.8	4.3 ± 0.8	4.7 ± 0.8
Volume (mL)	68.1 ± 19	60.3 ± 14	73.3 ± 20
Volume Indexed (mL/m^2^)	36.3 ± 7.8	36.1 ± 7.7	36.5 ± 7.8

^a^ All values are mean and standard deviation or proportions

We categorized our clinical patients into four groups based on their LAVi cutoff values:

- Normal – 21 to 52 mL/m^2^
- Mild – 52 to 62 mL/m^2^
- Moderate – 63 to 73 mL/m^2^
- Severe – greater than 73 mL/m^2^

Compared with healthy volunteer subjects, the clinical patient cohort was older and had a slightly higher BSA. Baseline characteristics of the patient population are described (Table 2). We found that LA enlargement was associated with older age, male gender, increasing prevalence of atrial fibrillation, history of hypertension, diagnosis of heart failure and increasing use of anticoagulants and antihypertensive medications such as renin-angiotensin-aldosterone inhibitors (e.g., angiotensin-converting enzyme inhibitors, angiotensin II receptor blockers and aldosterone receptor antagonists), beta blockers, nitrates, calcium channel blockers, and diuretics. Imaging parameters associated with increasing LA size included increasing prevalence of LGE (LV scar), dilated ventricles, and decreased LV and right ventricular (RV) EF. Asians were found to have smaller LAVi (Median 42.5, Interquartile Range [IQR] 34, 57.4) compared to Whites (Median 47.6, IQR 36.6, 62.8) (Wilcoxon rank-sum [WRS] Asian and White *P* < 0.001), Blacks (Median 46, IQR 35.7, 61.4) (WRS Asians and Black *P* = 0.014) or other races (Median 48.7, IQR 37.2, 64.2) (WRS Asians and Others *P* < 0.001).

**Table 2:** Baseline characteristics of patient population by LA enlargement

Variable ^a^	Overall (*n* = 10,890)	Normal [< 52 mL/m^2^] (*n* = 6471)	Mild [52–62 mL/m^2^] (*n* = 1617)	Moderate [63–73 mL/m^2^] (*n* = 1142)	Severe [> 73 mL/m^2^] (*n* = 1660)	*P* – value ^b^
Age (years)	60 (48, 69)	57 (44, 67)	61 (51, 70)	64 (54, 72)	65 (55, 75)	< 0.001
Female (%)	43%	46%	43%	39%	36%	< 0.001
Race						
White (%)	74%	73%	74%	76%	74%	0.09
Black (%)	17%	17%	16%	16%	17%	
Asian (%)	4%	5%	4%	3%	3%	
Other (%)	5%	5%	6%	5%	6%	
Anthropometric Indices						
BMI (kg/m^2^)	27.7 (24.2, 32)	28 (24.3, 32.3)	28 (24.4, 32.1)	27.8 (24.3, 31.6)	26.4 (23.4, 30.5)	< 0.001
BSA (m^2^)	2 (1.8, 2.2)	2 (1.8, 2.2)	2 (1.8, 2.2)	2 (1.8, 2.2)	2 (1.8, 2.1)	< 0.001
Systolic BP (mmHg)	127 (114, 140)	127 (115, 139)	127 (115, 141)	128 (114, 141)	126 (112, 141)	0.19
Diastolic BP (mmHg)	73 (65, 82)	73 (65, 82)	73 (64, 82)	72 (64, 81)	72 (64, 82)	0.3
Heart Rate (bpm)	72 (63, 82)	72 (63, 82)	70 (62, 81)	71 (62, 83)	73 (62, 85)	< 0.001
Hematocrit (%)	41 (37, 45)	42 (38, 45)	41 (36, 45)	40 (35, 44)	40 (36, 45)	< 0.001
eGFR (mL/min)	78 (62, 97)	81 (66, 101)	77 (60, 94)	74 (57, 90)	70 (56, 88)	< 0.001
Clinical History						
Myocardial Infarction (%)	14%	12%	15%	17%	16%	< 0.001
Current Smoker (%)	10%	10%	9%	10%	9%	0.66
Atrial fibrillation (%)	9%	4%	11%	16%	24%	< 0.001
Non-ischemic CMP (%)	9%	7%	10%	10%	15%	< 0.001
Heart Failure	25%	17%	28%	37%	45%	< 0.001
NYHA Class I (%)	6%	10%	3%	6%	3%	< 0.001
NYHA Class II (%)	36%	43%	35%	32%	29%	
NYHA Class III (%)	51%	41%	56%	56%	60%	
NYHA Class IV (%)	7%	6%	5%	6%	8%	
History of Hypertension (%)	63%	58%	67%	72%	70%	< 0.001
History of Dyslipidemia (%)	51%	49%	52%	53%	53%	0.007
History of Diabetes (%)	23%	22%	26%	27%	22%	< 0.001
Family History of CAD (%)	49%	47%	50%	54%	54%	< 0.001
Inpatient (%)	33%	28%	34%	39%	44%	< 0.001
Reason for Scan						
CHF/CMP Evaluation (%)	23%	13%	18%	25%	68%	< 0.001
Valve Assessment (%)	16%	15%	21%	23%	13%	< 0.001
Coronary Artery Disease (%)	19%	22%	22%	17%	9%	< 0.001
Arrhythmia (%)	12%	8%	9%	11%	33%	< 0.001
Congenital (%)	7%	9%	5%	5%	1%	< 0.001
Mass/Thrombus (%)	6%	7%	5%	3%	3%	< 0.001
Medication use						
Antihypertensive use (%)	77%	71%	81%	85%	89%	< 0.001
Antiplatelet use (%)	48%	47%	49%	52%	47%	0.013
Anticoagulant use (%)	23%	17%	23%	30%	37%	< 0.001
Statin use (%)	44%	42%	45%	48%	45%	0.006
Cardiac Magnetic Resonance Imaging Characteristics						
LA volume Indexed (mL/m^2^)	47 (36, 63)	39 (32, 45)	57 (54, 60)	67 (65, 70)	88 (79, 103)	–
LVEDV Indexed (mL/m^2^)	71 (56, 91)	64 (53, 79)	77 (62, 98)	83 (66, 106)	96 (71, 125)	< 0.001
LVESV Indexed (mL/m^2^)	27 (18, 44)	23 (16, 34)	30 (20, 51)	34 (22, 60)	44 (26, 75)	< 0.001
LVSV Indexed (mL/m^2^)	40 (32, 49)	39 (31, 46)	43 (34, 52)	43 (33, 53)	44 (33, 58)	< 0.001
LVEF (%)	61 (48, 69)	63 (53, 70)	60 (44, 69)	59 (40, 68)	54 (35, 66)	< 0.001
LV Mass (g)	131 (98, 174)	118 (91, 154)	144 (109, 190)	152 (115, 194)	166 (123, 211)	< 0.001
LV Cardiac Output (L/min)	5.6 (4.4, 6.9)	5.3 (4.2, 6.5)	5.8 (4.7, 7.3)	5.9 (4.7, 7.5)	6.2 (4.9, 8)	< 0.001
LV CO Indexed (L/min/m^2^)	2.9 (2.3, 3.5)	2.7 (2.2, 3.3)	3 (2.5, 3.6)	3 (2.4, 3.8)	3.2 (2.5, 4.1)	< 0.001
RVEDV Indexed (mL/m^2^)	69 (55, 86)	64 (51, 78)	72 (58, 88)	77 (62, 93)	83 (65, 106)	< 0.001
RVESV Indexed (mL/m^2^)	32 (24, 43)	29 (22, 38)	33 (25, 44)	37 (28, 49)	44 (31, 63)	< 0.001
RVSV Indexed (mL/m^2^)	35 (27, 44)	35 (27, 43)	38 (29, 46)	38 (29, 46)	36 (28, 46)	< 0.001
RVEF (%)	53 (45, 60)	54 (48, 61)	54 (45, 60)	51 (42, 58)	47 (36, 55)	< 0.001
LV Myocardial Scar (%)	0 (0, 3)	0 (0, 2)	0 (0, 4)	0 (0, 5)	1 (0, 5)	< 0.001
Any Scar in LV (%)	38%	32%	42%	45%	53%	< 0.001
Outcomes						
Death	835 (7.7%)	394 (6.1%)	133 (8.2%)	103 (9%)	205 (12.4%)	< 0.001

*BMI* body mass index, *BSA* body surface area, *BP* blood pressure, *eGFR* estimated glomerular filtration rate, *CHF* congestive heart failure, *CMP* cardiomyopathy, *LA* left atrial, *LV* left ventricular, *RV* right ventricular, *EDV* end diastolic volume, *ESV* end systolic volume, *SV* stroke volume, *EF* ejection fraction, *CO* cardiac output

^a^ All values are median (interquartile ranges) and proportions

^b^ *P* -values are based on Kruskal-Wallis (continuous variables) and χ^2^ test (categorical variables)

### Clinical outcomes

There were 835 (7.7%) all-cause mortality events in the total cohort. There was a significant increase in the prevalence of mortality with increasing severity of LAVi enlargement (Normal: 6.1% [394/6471], Mild: 8.2% [133/1617], Moderate: 9% [89/1142], Severe: 12.4% [241/1660]) (*P* < 0.001).

**Fig. 3:**
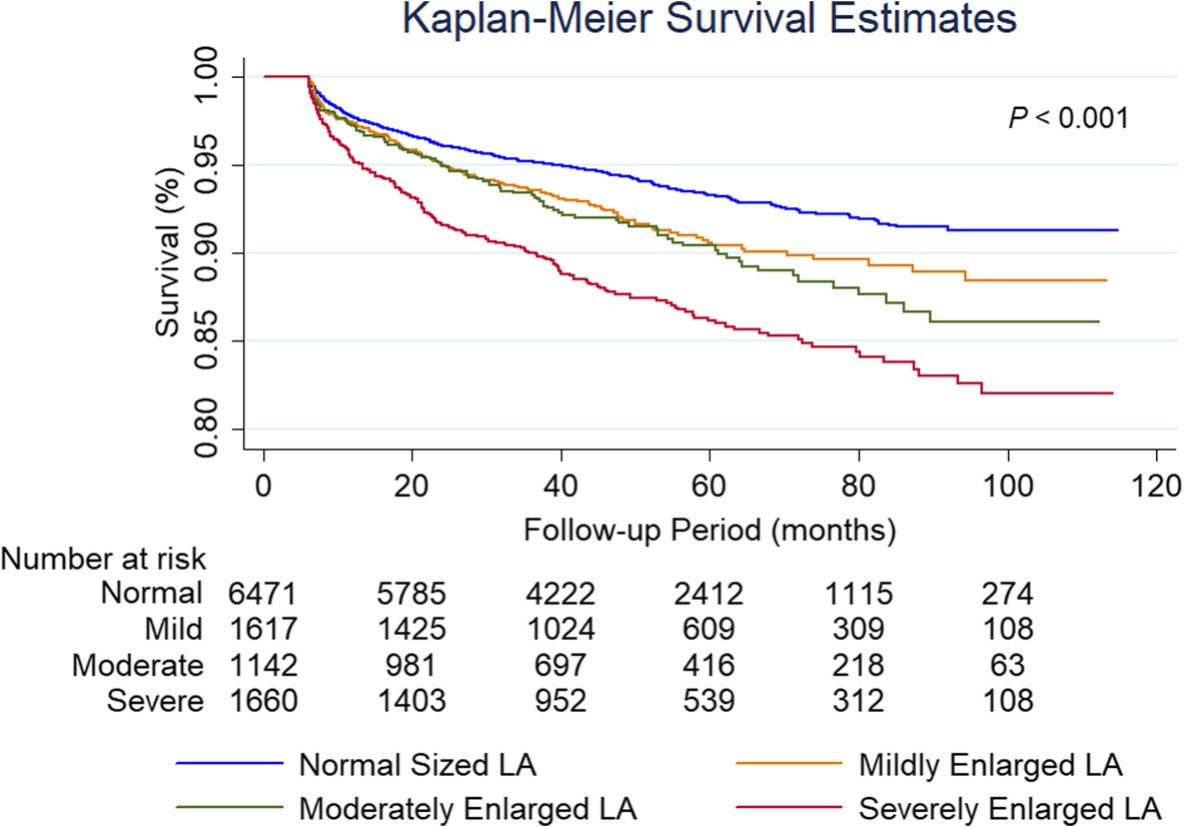
Time to event Kaplan-Meier curve presenting the association of all-cause mortality with increasing LAVi. Normal = left atrial volume indexed: 21 to 52 ml/m^2^; Mild = 52 to 62 ml/m^2^; Moderate = 63 to 73 ml/m^2^; Severe = greater than 73 ml/m^2^

**Fig. 4:**
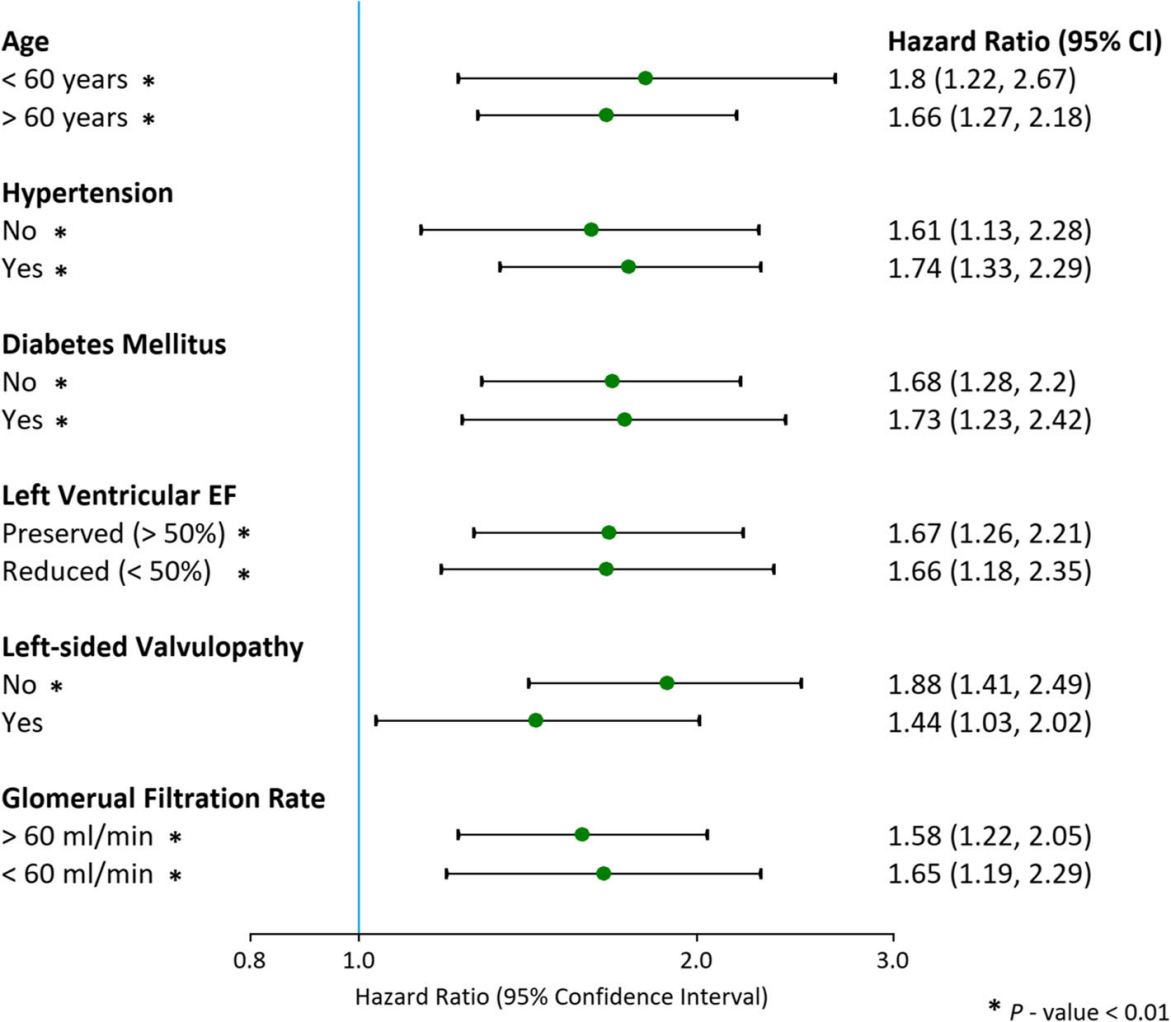
Risk of all-cause mortality in patients with severe LA enlargement in various sub-groups. Hazard ratios for each sub-group were calculated using multivariate models. Age < 60 = *adjusted for hypertension, LVEF, LV scar*; Age > 60 = *adjusted for hypertension, diabetes mellitus, atrial fibrillation, LVEF, RVEF, LV mass, LV scar*; Non-hypertensive = *adjusted for age, diabetes mellitus*; Hypertensive = *adjusted for age, atrial fibrillation, diabetes mellitus, myocardial infarction, LVEF, RVEF, LV mass, LV scar*; Non-diabetic = *adjusted for age, hypertension, atrial fibrillation, LVEF, RVEF, LV scar*; Diabetic = *adjusted for age, hypertension, LVEF*; Preserved LVEF (> 50%) and Reduced LVEF (< 50%) = *adjusted for age, hypertension, diabetes mellitus, RVEF, LV scar*; No Left-sided Valvulopathy = *adjusted for age, hypertension, diabetes mellitus, LVEF*; Left-sided Valvulopathy = *adjusted for age, hypertension, LVEF*; eGFR > 60 ml/min = *adjusted for age, hypertension, diabetes mellitus, LVEF, LV scar*; eGFR < 60 ml/min = *adjusted for age, hypertension, diabetes mellitus, LVEF*

**Fig. 5:**
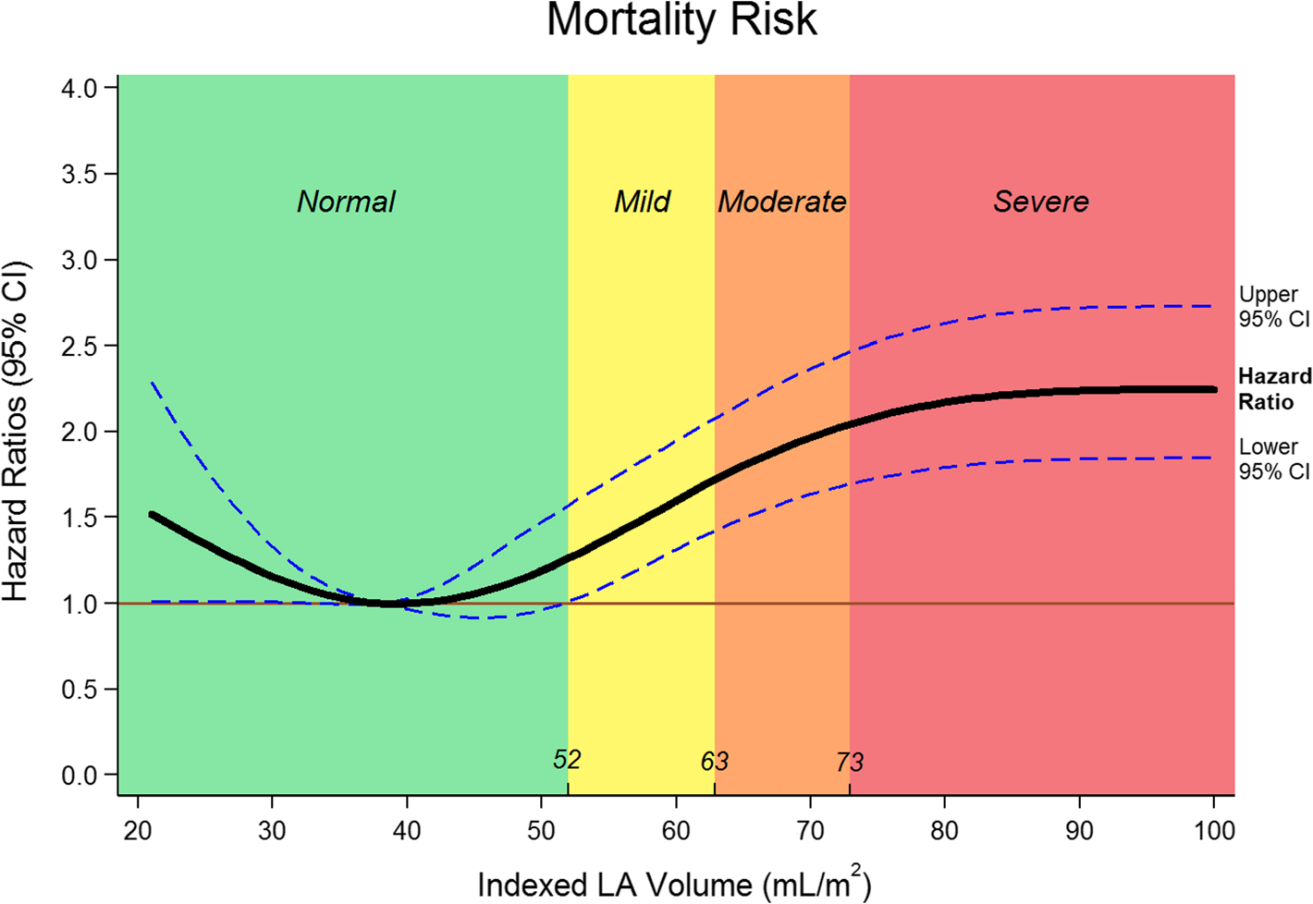
Hazard ratios for LAVi as a continuous variable. Restricted cubic spline model of hazard ratios for left atrial volume indexed. The 3 curves represent the hazard ratio and the upper and lower bounds of the 95% confidence interval at any given LAVi. The transition point of the lower bound of the 95% confidence interval occurs at 52 ml/m^2^ which also correspond to the upper limit of normal LA size derived by standard deviations. Hazard ratios after 73 ml/m^2^ tend to even out as the gradient of the curve plateaus

On univariate analysis, older age, BMI, lower systolic and diastolic blood pressure, faster heart rate, lower eGFR, inpatient hospitalization status, history of hypertension, history of diabetes mellitus, history of dyslipidemia, prior myocardial infarction, decreased indexed LV stroke volume, decreased LV EF, increased LV mass, larger LV scar and decreased RV EF were significant predictors of mortality. Mild (hazard ratio [HR] 1.35, [95% CI 1.11, 1.65; *P* = 0.003), moderate (HR 1.51, [95% CI 1.22, 1.88]; *P* < 0.001) and severe (HR 2.14, [95% CI 1.81, 2.53]; *P* < 0.001) LA enlargement were robust predictors of mortality (Fig. 3). Even after adjusting for clinically relevant covariates (Model 2), LA enlargement remained significant predictor of mortality. After the addition of CMR imaging variables to the model (Model 3), only moderate LA enlargement (HR 1.45, [95% CI 1.1, 1.89]; *P* = 0.006) and severe LA enlargement (HR 1.64, [95% CI 1.29, 2.08]; *P* < 0.001) remained significant predictors of mortality (Table 3). Atrial fibrillation (HR 0.77, [95% CI 0.57, 1.03]; *P* = 0.08), history of hypertension (HR 1.003, [95% CI 0.1, 1.24]; *P* = 0.98) and LV mass (HR 1.001, [95% CI 0.999, 1.003]; *P* = 0.19) did not show significance on the multivariate analysis (Model 3). Severe LA enlargement remained an independent predictor of mortality in various subgroup analyses (Fig. 4). For the analysis of LAVi as a continuous variable using restricted cubic spline regression model, a baseline value of 38 ml/m^2^ was selected. This value was derived by calculating the mean value of LAVi for the “Normal” group. As a continuous variable, every 5 ml increase in LAVi was associated with increasing odds of mortality (Fig. 5) on the univariate (HR 1.01, [95% CI 1.002, 1.01]; *P* < 0.001) and multivariate analysis (Model 2: HR 1.004 [95% CI 1.002, 1.01]; *P* = 0.001; Model 3: HR 1.004 [95% CI 1, 1.01]; *P* = 0.046).

**Table 3:** Association of severity of LA enlargement and all-cause mortality

Variable	Model 1		Model 2		Model 3	
	Hazards ratio (95% CI)	*P* - value	Hazards ratio (95% CI)	*P* - value	Hazards ratio (95% CI)	*P* - value
Normal LA size	1.00	–	1.00		1.00	–
Mild LA enlargement	1.35 (1.11, 1.65)	0.003	1.27 (1.04, 1.55)	0.022	1.04 (0.78, 1.36)	0.76
Moderate LA enlargement	1.51 (1.22, 1.88)	< 0.001	1.32 (1.05, 1.66)	0.017	1.45 (1.1, 1.89)	0.006
Severe LA enlargement	2.14 (1.81, 2.53)	< 0.001	1.9 (1.58, 2.28)	< 0.001	1.64 (1.29, 2.08)	< 0.001
LAVI (for every 5 ml increase)	1.01 (1.002, 1.01)	< 0.001	1.004 (1.002, 1.01)	0.001	1.004 (1, 1.01)	0.046

Model 1 – Univariate analysis

Model 2 – Adjusted for clinical variables; age, gender, atrial fibrillation, history of hypertension, history of diabetes and history of myocardial infarction

Model 3 – Model 2 + adjusted for CMR variables; left ventricular ejection fraction, left ventricular mass, left ventricular scar, right ventricular ejection fraction

## Discussion

Prior normal LA reference values calculated by CMR used the 3D mapping technique requiring a stack of short-axis stack cines through the LA, or a biplane area-length method which included the LA appendage. Our study derived the LA reference range using biplane area-length method excluding the appendage in a U.S. based cohort of healthy volunteer subjects without known CVD. This method allowed us to use values that were acquired using standard clinical 4- and 2-chamber LV views, without the need of extra acquisitions that would necessitate a longer study duration and extra breath-holds, which can be challenging for some clinical patients. Our normal values did not significantly differ from LA volumes measured by 3D techniques [20]. For our second objective, we measured the association of LA enlargement with all-cause mortality in our multicenter clinical patient cohort. Our study cohort included patients referred for CMR from 3 different geographic sites. We categorized our patients into groups with increasing LA size using the risk of all-cause mortality derived from ROC analyses. We demonstrate a robust association of increasing LA size and all-cause mortality, even after adjustment for other dominant predictors of mortality such as age, history of hypertension, history of diabetes mellitus, history of myocardial infarction, LVEF, RVEF, and LV scar.

There is a well-known association between LA enlargement and all-cause mortality in both the general population and high risk patient cohorts (those with heart failure and stroke) [14, 15, 27–29]. However, all prior studies have been based on echocardiographic data, which have different reference values compared to CMR [30, 31]. Our study is consistent with the results of prior echocardiographic studies showing the association between LAVi and mortality. We used a patient population referred for CMR without excluding any co-morbidities like valvular heart disease, depressed LVEF, or malignancy. This allowed us to assess the significance of LA enlargement in a very large, heterogeneous clinical patient cohort.

The numerous mechanisms and pathways of LA enlargement have not been completely understood. Some of the most common causes of an enlarged LA are mitral and aortic valve disorders [1, 2], hypertensive heart disease [32], depressed LV systolic [3] and diastolic function [4]. The mechanism of LA enlargement in these pathologies could be attributed to chronically maintained volume overload or elevated left atrial pressure, resulting in LA remodeling. Although LA size is often considered a surrogate marker of chronically elevated LV filling pressure, LA volumes in patients on optimal medical therapy, including diuretics; may reflect effective medical therapy and mask the severity of impaired LV performance. When assessing the association of LA enlargement and all-cause mortality, the mechanism accounting for outcomes is not fully understood. This effect may be a representation of the underlying pathologies causing elevated LV filling pressure, or may be attributed to arrhythmias, most often atrial fibrillation which is often a result of left atrial enlargement [7]. This could explain the higher incidence of embolic events, heart failure hospitalizations and all-cause mortality in patients with large left atria. It is, nevertheless, difficult to evaluate whether the atrial fibrillation is caused by an enlarged left atria or vice versa [33].

### Limitations

Our study had limitations. The patient population selected for the study had at least 1 clinical indication for CMR. This introduces a selection bias of having a relatively symptomatic group of people as the study cohort, in comparison to a general population. The data we gathered through CloudCMR may not have included all clinically relevant variables. Hence not all significant variables, such as biomarkers of increased mechanical load and wall stretch, were available on all patients. However, such biomarkers are not routinely assessed in the outpatient setting. LA volumes measured in the patient cohort was derived at the time of clinical reporting by board certified physicians from 3 geographically distinct institutes. This could potentially be a source of discrepancy and bias in LAVi measurement. LA volumes were measured using the biplane area-length method, which is not the gold-standard method of measuring LA volume. There is the potential for foreshortening which could have affected the LA volume calculation. However, we feel that the cost of accuracy is compensated for by practicality, as this method can be performed on essentially all clinical cine-CMR studies. Approximately 9% of the patient population had atrial fibrillation at the time of our scan, in which case prospective triggering was commonly utilized to acquire cine images. Although cine image quality can be affected in patients with arrhythmias, the ventricular end systolic phase was always captured and hence may not affect the measurement of maximum LA volume significantly. Our database was constructed from patients from 3 different sites, with each site having multiple CMR technologists and board-certified level 3 physician readers, which could introduce heterogeneity in measuring techniques of LA volumes. Nevertheless, this aspect of our study supports the external validity and generalizability of our findings. The number of death events recorded in CloudCMR was reliant on the electronic health records and SSDI. This could slightly underreport the actual number of deaths in the cohort due to an absence of direct patient contact and status verification. Lastly, the CloudCMR database did not have the capability to capture clinical outcomes other than death at the time of our query. Therefore, we were unable to investigate the association of LA size with heart failure events or other cardiovascular endpoints.

## Conclusion

CMR is considered a gold standard technique at measuring cardiac chamber volumes. We showed LA enlargement measured by routinely performed 4- and 2- chamber cine-CMR images demonstrates a strong independent association with all-cause mortality. We also established LA enlargement classification by standard deviation method correlated precisely with risk of mortality. Further studies are needed to supplement the classification of LA enlargement severity using CMR, based off of data observing mortality, heart failure admissions, and other cardiovascular events.

## Abbreviations

ASE: American Society of Echocardiography
BSA: Body surface area
bSSFP: Balanced, steady state free precession
CMR: Cardiovascular magnetic resonance
CVD: Cardiovascular disease
EACVI: European Association of Cardiovascular Imaging
EF: Ejection fraction
eGFR: Estimated glomerular filtration rate
HR: Hazard ratio
ICC: Intraclass correlation coefficients
IQR: Interquartile range
LA: Left atrium/left atrial
LAVi: Left atrial volume indexed to body surface area
LGE: Late gadolinium enhancement
LV: Left ventricle/left ventricular
ROC: Receiver operator curve
RV: Right ventricle/right ventricular
SSDI: Social Security Death Index
